# Challenges in measuring acceptability, appropriateness, and feasibility: a case study in the pediatric intensive care unit

**DOI:** 10.1186/s43058-026-00967-2

**Published:** 2026-05-29

**Authors:** Amanda O’Halloran, Robert M. Sutton, Meghan Lane-Fall

**Affiliations:** 1 Department of Anesthesiology and Critical Care Medicine, University of Pennsylvania Perelman School of Medicine, 3401 Civic Center Boulevard, Office 9NW103, Philadelphia, PA 19104 USA; 2 Department of Anesthesiology and Critical Care, The Children’s Hospital of Philadelphia, Philadelphia, PA USA; 3 Leonard Davis Institute of Health Economics, University of Pennsylvania, Philadelphia, PA USA; 4 Department of Anesthesiology, Columbia University Vagelos College of Physicians and Surgeons, New York, NY USA; 5 New York-Presbyterian/Columbia University Medical Center, New York, NY USA

**Keywords:** Acceptability, Appropriateness, Feasibility, Implementation outcomes

## Abstract

**Background:**

Rigorous development and evaluation of implementation outcome measures is needed to advance the field of implementation science. The Acceptability of Intervention Measure (AIM), Feasibility of Intervention Measure (FIM), and the Intervention Appropriateness Measure (IAM) are commonly used, psychometrically validated measures. The objective of the current analysis was to analyze use of the AIM, FIM, and IAM in a single-center pediatric intensive care (PICU) unit study.

**Methods:**

This was a secondary analysis of a mixed-methods interventional study. The initial project studied development of a novel cardiac arrest ventilation rate metronome to improve adherence to guideline-recommended ventilation rates during CPR; study components included contextual inquiry, participatory intervention design, and simulation usability testing. Using a purposive sampling strategy for pre-implementation contextual inquiry, eligible participants included multidisciplinary PICU clinicians. The pre-implementation questionnaire asked about current cardiac arrest ventilation practices and perceived acceptability, appropriateness, and feasibility of our proposed intervention (AIM, FIM, and IAM). Five-point scale Likert data were summarized using descriptive statistics (medians and interquartile ranges). A score of > 3 was considered favorable.

**Results:**

Of 133 started instances of the pre-implementation survey, 107 were completed (80.5%). The response rate was 30.6% (107/350). Respondents included 34 nurses (31.8%), 18 respiratory therapists (16.8%), and 55 ordering providers (physicians and nurse practitioners; 51.4%). Most respondents (79/107; 73.8%) had previously participated in > 10 PICU cardiac arrests. Appropriateness, acceptability, and feasibility of the ventilation metronome were favorable (appropriateness median: 4 [IQR 4,5]; acceptability median: 4 [IQR 3,5]; feasibility median: 4 [IQR 4,4.5]). For each AIM, IAM, and FIM statement, ≥24.3% of all responses were the highest value on the 5-point scale. Of the 19.5% of survey instances with only partial responses, all but one (26/27; 96.3%) were stopped at the AIM, IAM, FIM matrix of statements.

**Conclusions:**

In this single-center PICU study utilizing the previously validated Acceptability of Intervention Measure, Feasibility of Intervention Measure, and Intervention Appropriateness Measure, there were several challenges associated with the use of these scales, including (1) survey responses that were terminated at the portion of the questionnaire presenting the AIM, IAM, and FIM and (2) ceiling effect.

**Supplementary Information:**

The online version contains supplementary material available at 10.1186/s43058-026-00967-2.

Text box 1. Contributions to the literature

- Novel contextual descriptive analysis of the performance of psychometrically validated implementation measures of acceptability, appropriateness, and feasibility (Acceptability of Intervention, Intervention Appropriateness, and Feasibility of Intervention Measures) in a pediatric intensive care unit study.
- Limitations to the use of these measures included: (1) ceiling effect and (2) of those surveys with partial responses, the majority were terminated at these implementation measures.
- Our results suggest that context-specific adaptations and considerations are required to ensure optimal application of implementation measures.

## Introduction

While core implementation outcomes have been established, rigorous development and evaluation of measures of these outcomes continue to be needed to advance the field of implementation science [1–3]. Specifically, in addition to psychometric validation, context-specific evaluation of how measures function in practice within particular clinical settings and for particular interventions would help implementation researchers choose the optimal measure for the intervention in question. Such evaluation can help determine whether a measure is usable, acceptable to respondents, and able to generate meaningful and interpretable data in a real-world implementation context.

Of the eight core implementation outcomes, acceptability, appropriateness, and feasibility are among the most commonly reported in the literature [3]. In 2017, Weiner et al. performed a rigorous psychometric validation of measures of acceptability, appropriateness, and feasibility: the Acceptability of Intervention Measure (AIM), Feasibility of Intervention Measure (FIM), and the Intervention Appropriateness Measure (IAM) [4]. While these measures have been cited in many implementation research studies and protocols since 2017, context-specific evaluation of their use is limited to date. In this sense, such evaluation can be understood as a process-oriented examination of how these measures operate in actual use within a specific setting.

To that end, our team conducted a detailed analysis of the use of the previously validated measures of acceptability, appropriateness, and feasibility (the AIM, IAM, and FIM) in a single-center pediatric intensive care unit (PICU) study [4].

## Methods

This study was undertaken in the Children’s Hospital of Philadelphia (CHOP) PICU, which is a 75-bed medical-surgical unit. Clinicians of various disciplines who routinely care for children in the CHOP PICU were recruited to participate in this study, including bedside nurses, nurse practitioners, physicians, physician assistants, and respiratory therapists.

To improve adherence to guideline-recommended ventilation rates during pediatric cardiopulmonary resuscitation (CPR) with an advanced airway in place, implementation science and human factors engineering were utilized to design and implement a novel digital audiovisual cardiac arrest ventilation rate metronome. This metronome, along with an explicit discussion of the goal ventilation rate for each patient during CPR, comprised a CPR ventilation rate improvement bundle that has since been implemented in the CHOP PICU. The study framework was based on the Systems Engineering Initiative for Patient Safety (SEIPS) 2.0 combined with a breakdown of the work system into relevant macroergonomic subsystems [5, 6]. Components of the study, which were explained in detail in a previous publication, are described briefly below [7]. The study was reviewed by the CHOP Institutional Review Board and determined to be exempt (IRB 21-019048). This manuscript reports a secondary analysis of cross-sectional survey data, and we followed the Strengthening the Reporting of Observational Studies in Epidemiology (STROBE) guidelines in preparing the report [8].

### Description of development of the cardiac arrest ventilation rate metronome

First, a clinical consensus conference was held to ensure consensus on the unit’s standard target ventilation rates during CPR with an advanced airway.

Second, multifaceted contextual inquiry with a pragmatic paradigm was undertaken to gain an understanding of the perspectives of impacted parties regarding the proposed ventilation improvement bundle. Family caregiver perspectives were elicited during a session with the CHOP Research Institute’s Family Advisory Council. As part of this pre-implementation contextual inquiry phase, we administered a survey to CHOP PICU clinicians before the ventilation bundle was finalized and prior to simulation-based iterative design and usability testing. The survey was used to assess clinicians’ perspectives on current cardiac arrest ventilation practices and their anticipated perceptions of the proposed bundle, including its acceptability, appropriateness, and feasibility. We surveyed CHOP PICU clinicians for their opinions on: (1) current cardiac arrest ventilation delivery practices and (2) our proposed bundle to improve ventilation during cardiac arrest. Additional detail related to this survey, which is the focus of this secondary analysis, is included below.

Third, multidisciplinary simulation-based iterative design sessions were held to inform development of the cardiac arrest ventilation rate metronome. Once consensus was reached on a final design, high-fidelity simulation usability testing was conducted (February 2024) until thematic saturation was reached. Team composition, room set-up, and the simulation scenario were designed to mimic actual CHOP PICU resuscitations as closely as possible. Data collection included narrative participant feedback, System Usability Scale scores [9], direct observations by the study team, and extraction of resuscitation process metrics (including ventilation rate) from video review of the scenarios.

### Pre-implementation PICU clinician survey

Between April and June 2023, we surveyed CHOP PICU clinicians’ perspectives on current PICU cardiac arrest ventilation practices and the acceptability, feasibility, and appropriateness of the proposed ventilation bundle. This survey was deployed during the pre-implementation phase of the project as part of the contextual inquiry used to inform intervention development. Respondents were asked to rate the proposed intervention based on a description of the proposed ventilation bundle provided during the clinical consensus conference and their understanding of how it would be used during PICU cardiac arrest care, rather than on direct experience using the bundle in clinical care or simulation. The questionnaire included the previously validated Acceptability of Intervention Measure [AIM], Feasibility of Intervention Measure [FIM], and Intervention Appropriateness Measure [IAM] scales [4]. The survey began with 3 items about current ventilation practices, followed by a brief reminder/description of the proposed ventilation bundle and the 12 AIM, IAM, and FIM statements presented as a matrix. The remaining items included 1 conditional free-text question about implementation barriers and 3 questions about clinician discipline, prior cardiac arrest experience, and cardiac arrest roles (**Supplement**). The five-point scale Likert data were summarized using descriptive statistics (medians and interquartile ranges). A score of > 3 was considered favorable since responses of 4 and 5 correspond with ‘agree’ or ‘completely agree,’ respectively. Extracted free-text responses were mapped to the study framework’s macroergonomic subsystems such as personnel, technical, and internal environment domains. These qualitative findings informed the broader parent study and have been reported previously; they are not a focus of the present secondary analysis [7].

Clinicians who would be directly impacted by implementation of the ventilation bundle were eligible to participate, including CHOP PICU nurses, nurse practitioners, physicians, physician assistants, and respiratory therapists. Utilizing a purposive sampling strategy, invitations to complete the questionnaire were emailed to all eligible participants. We aimed to achieve a 30% response rate. No incentives were provided for participating. The electronic questionnaire was administered to CHOP PICU clinicians via Research Electronic Data Capture, which is a secure, web-based software platform designed to support data capture for research studies that has been described previously [10, 11]. Responses were collected and stored anonymously on a secure CHOP network location.

## Results

There were an estimated 350 eligible PICU clinicians. One hundred thirty-three instances of the pre-implementation survey were started (38% response rate; 133/350). 107 survey responses were completed (80.5%; 107/133). Survey respondent characteristics are summarized in Table 1. Respondents included 34 nurses (31.8%), 18 respiratory therapists (16.8%), and 55 ordering providers (physicians and nurse practitioners; 51.4%). Most respondents (79/107; 73.8%) reported having participated in > 10 PICU cardiac arrests.

**Table 1:** Characteristics of survey respondents

Characteristic	Number (%) *n* = 107
**Discipline**	
Ordering clinicians	55 (51.4%)
Nurse practitioner	10 (9.3%)
Physician	45 (42.1%)
Physician assistant	00 (0%)
Nurse	34 (31.8%)
Respiratory therapist	18 (16.8%)
**Previous Participation in PICU Cardiac Arrests**	
0	5 (4.7%)
1–5	10 (9.3%)
6–10	13 (12.1%)
More than 10	79 (73.8%)
**Cardiac Arrest Roles (select all that apply)**	
Chest compressions	84 (78.5%)
Medication administration	73 (68.2%)
Providing ventilations	51 (47.7%)
CPR coach	46 (43%)
Event leader	43 (40.2%)
Use of defibrillator	38 (35.5%)
Documentation	33 (30.8%)
Other respiratory therapy responsibilities*	22 (20.6%)
Other	21 (19.6%)

*This may include tasks such as intubation equipment preparation, initiating endtidal carbon dioxide monitoring, etc

Appropriateness, acceptability, and feasibility of the ventilation metronome were favorable with median scores of 4 for each outcome: appropriateness 4 (IQR 4,5), acceptability 4 (IQR 3,5), and feasibility 4 (IQR 4,4.5) (Table 2). For each of the 12 statements comprising these 3 measures, ≥24.3% of all responses were the highest possible value on the 5-point Likert scale (completely agree; range of 24.3%-33.6%). Supplemental Fig. 1 shows the distribution of Likert responses across the IAM, AIM, and FIM, demonstrating that responses for all three measures were concentrated in the “Agree” and “Completely agree” categories. Of the 19.5% of instances of the survey with only partial responses, all but one (26/27; 96.3%) were stopped at the matrix of statements measuring acceptability, appropriateness, and feasibility. Because this matrix appeared near the beginning of the brief survey, after only 3 initial items on current ventilation practices and a brief description of the proposed intervention, most partial responses terminated before respondents reached the later free-text and respondent-characteristic items.

**Table 2:** Measurement of appropriateness, acceptability, and feasibility of proposed intervention among PICU clinicians

Measure	Clinician Response (median [IQR]) *n* = 107
Appropriateness of Proposed Intervention*	**4 (4, 5)**
The ventilation bundle seems fitting.	4 (4, 5)
The ventilation bundle seems suitable.	4 (4, 5)
The ventilation bundle seems applicable.	4 (4, 5)
The ventilation bundle seems like a good match.	4 (4, 5)
Acceptability of Proposed Intervention*	**4 (3.5**,** 5)**
The ventilation bundle meets my approval.	4 (4, 5)
The ventilation bundle is appealing to me.	4 (3, 5)
I like the ventilation bundle.	4 (3, 5)
I welcome the ventilation bundle.	4 (4, 5)
Feasibility of Proposed Intervention*	**4 (4**,** 4.5)**
The ventilation bundle seems implementable.	4 (4, 4)
The ventilation bundle seems possible.	4 (4, 5)
The ventilation bundle seems doable.	4 (4, 5)
The ventilation bundle seems easy to use.	4 (4, 4)

All responses are on a 5-point Likert scale, with ratings from 1 (Never/Completely Disagree) to 5 (Always/Completely Agree)

*Median of the items included in that specific measure. IQR=interquartile range

## Discussion

In this single-center PICU study of the development of an intervention to improve cardiac arrest care, acceptability, appropriateness, and feasibility were assessed as anticipated perceptual implementation outcomes of impacted clinicians [12, 13]. To our knowledge, this is the first published analysis specifically of the performance of these measures in an intensive care setting and our results suggest that there may be context-specific considerations for their use that require additional investigation. We noted that nearly 20% of instances of our survey received only partial responses, and of those, 96.3% (26/27) were stopped at the matrix of 12 statements comprising the AIM, IAM, and FIM scales. Moreover, the median scores for each measure *and* for each individual statement within the three measures were all 4, with a large proportion of respondents (24.3%-33.6%) selecting the highest value on the 5-point Likert scale.

Our study team noted only a moderately high partial response rate to our pre-implementation survey, but with a very high (96.3%) proportion of the partial responses that were stopped at the AIM, IAM, and FIM scales. This pattern is notable because the 12-item AIM, IAM, and FIM matrix appeared near the beginning of a relatively brief survey, after only 3 prior items and a short reminder of the description of the proposed intervention. We propose several possible explanations for this, with specialty- and unit-specific contextual factors that may contribute [14–16]. First, while these measures are psychometrically validated, they may not be engaging or appealing to our surveyed population of ICU clinicians. Of note, there have been multiple previous surveys completed in our unit that have also used the AIM, IAM, and FIM scales [17]. Although their repeated use reflects the unit’s commitment to standardized implementation outcomes, it may also mean that clinicians have been exposed to these same 12 statements many times. As a result, some clinicians may experience response fatigue or diminished interest. Second, the measures may be poorly suited to the intervention in question—a highly technical metronome for use in cardiac arrests. Third, utilizing all three measures, and therefore 12 statements, may have increased the length sufficiently to make the time required to complete the questionnaire a barrier for more clinicians. While the dataset from this study did not explore these hypothesized reasons, future studies could interrogate target populations’ context-specific perspectives on these measures.

For all AIM, IAM, and FIM statements, a large proportion of survey respondents (24.3%-33.6%) selected the highest value on the 5-point Likert scale. With this somewhat narrow distribution of data, interpretation of positive responses was limited by the well-described ‘ceiling effect’ phenomenon [18, 19]. When a substantial proportion of respondents cluster at the top end of the scale, meaningful distinctions between strongly positive and moderately positive responses can be obscured. One possible explanation for this pattern is that clinicians genuinely viewed the proposed ventilation bundle favorably. In the broader parent study, complementary qualitative data suggested generally positive perceptions of the intervention, but also identified specific concerns and design considerations raised by clinicians [7]. This suggests that the high-end clustering observed in the AIM, IAM, and FIM could reflect both true positivity toward the intervention and a reduced ability of the scales to capture important nuance within those positive perceptions.

Particularly when these measures are being utilized prior to or early in adoption, greater differentiation in responses may be useful for informing intervention refinement. However, because our survey was administered only once during the pre-implementation phase, we could not examine whether or how these measures might perform if used longitudinally to inform iterative adaptation. Moreover, there are no clear best practices, to our knowledge, regarding repeated deployment of the AIM, IAM, and FIM for this purpose, and repeated administration in the same setting could itself worsen response burden or survey fatigue. While we are unaware of any studies adapting the response scale for the AIM, IAM, and FIM scales, potential strategies to mitigate ceiling effects could include increasing the number of response options, using positive-packed response scales, anchoring the highest response option more explicitly (e.g., “strong endorsement with no reservations”), supplementing numeric ratings with behavioral intention items, or incorporating open-ended prompts.

This report should be interpreted in light of its limitations. First, the reasons for partial survey responses could not be elucidated in this dataset. Although most incomplete responses terminated at the AIM, IAM, and FIM matrix, we cannot determine whether this reflected the content, format, or length of that section, or other respondent-specific factors. Second, we were unable to assess whether survey incompletion differed by clinician discipline because the discipline item appeared after the AIM, IAM, and FIM section, and all incomplete responses were therefore missing discipline data. Third, because all responses were collected anonymously, we cannot confirm that respondents of partial responses did not also later complete the entire survey. Fourth, because the survey was administered only once during the pre-implementation phase, this study could not assess whether repeated deployment of the AIM, IAM, and FIM would be useful for guiding iterative refinement of the intervention. There are no clear best practices, to our knowledge, for how or when to redeploy these measures for that purpose, and repeated administration could itself worsen response burden or survey fatigue. Fifth, clinicians evaluated the proposed ventilation bundle before direct use in simulation or clinical care. As a result, AIM, IAM, and FIM responses reflected anticipated perceptions based on the description of the intervention rather than experience with its actual use. Limited exposure to the intervention may have affected how accurately respondents could judge its acceptability, appropriateness, and feasibility, and some highly positive responses may have reflected general openness to the concept of the bundle rather than informed judgments about its performance in practice. Finally, generalizability is limited, as this was a single-center study of a large academic PICU with an approximately 30% response rate among eligible clinicians. However, this remains an important contribution to the literature that highlights the need for contextual study of implementation outcome measures.

## Conclusions

In this single-center PICU study utilizing the previously validated Acceptability of Intervention Measure, Feasibility of Intervention Measure, and Intervention Appropriateness Measure, there were several potentially context-specific challenges associated with the use of these scales, including survey responses that were terminated at the portion of the questionnaire presenting the AIM, IAM, and FIM and ceiling effects. These findings suggest that contextual inquiry may be useful not only for intervention development, but also for assessing how implementation outcome measures perform in a given clinical setting. Incorporating contextual inquiry into measure selection and evaluation may help researchers better anticipate challenges related to usability, respondent burden, and interpretability. Further investigation is needed to understand optimal use of these measures as their inclusion in implementation research becomes more widespread.

## Supplementary Information

Below is the link to the electronic supplementary material.

Supplementary Material 1

## Data Availability

The datasets used and/or analyzed during the current study are available from the corresponding author on reasonable request.
