# Supplementary material for: Challenges in measuring acceptability, appropriateness, and feasibility: a case study in the pediatric intensive care unit
Source: Implement Sci Commun. 2026 May 29;7:150. doi: 10.1186/s43058-026-00967-2 (PMC13471310; doi:10.1186/s43058-026-00967-2)
Supplement: Supplementary file 1 [file 43058_2026_967_MOESM1_ESM.docx]

**SUPPLEMENTAL CONTENT**

**Appendix 1. Pre-Implementation Survey**

Please review the consent form included with your email invitation prior to completing this survey. Completion of the survey indicates that you have read the consent form and have voluntarily agreed to participate.

**For each of the following 3 statements about ventilation delivery during cardiac arrests in the PICU, select the response that best describes your experience.**

1. I know the team’s ventilation rate goal for a particular patient during cardiac arrest.
2. A ventilation rate goal is discussed for each patient during a cardiac arrest.
3. The CHOP PICU team provides ventilation during cardiac arrest at a rate that is compliant with American Heart Association guidelines.

- All the time
- Most of the time
- Sometimes
- Rarely
- Never
- Don’t know
- This task is not relevant to my role(s)

**Our research team will be trialing an intervention that could improve ventilation delivery during cardiac arrest. The intervention (referred to as the *ventilation bundle*) will include:**

1. **A standardized script to discuss the ventilation rate goal and**
2. **The use of an audiovisual metronome set to that decided-upon goal.**

**For each of the following statements, select the response that best characterizes your feelings on this intervention.**

1. The ventilation bundle seems fitting.
2. The ventilation bundle seems suitable.
3. The ventilation bundle seems applicable.
4. The ventilation bundle seems like a good match.
5. The ventilation bundle meets my approval.
6. The ventilation bundle is appealing to me.
7. I like the ventilation bundle.
8. I welcome the ventilation bundle.
9. The ventilation bundle seems implementable.
10. The ventilation bundle seems possible.
11. The ventilation bundle seems doable.
12. The ventilation bundle seems easy to use.

- Completely Disagree
- Disagree
- Neither Agree Nor Disagree
- Agree
- Completely Agree

**If Disagree or Completely disagree were selected for any of the above questions:**

**What barriers do you think there would be to implementing the ventilation bundle?**

Free text response

**Please select your discipline.**

- Physician
- Nurse Practitioner
- Nurse
- Respiratory Therapist
- Physician Assistant
- Pharmacist
- SNA

**How many times have you participated in caring for a patient during cardiac arrest in the CHOP PICU?**

- 0
- 1-5
- 6-10
- More than 10

**Which of the following roles have you had or could you have during a cardiac arrest in the PICU (select all that apply)?**

- Event leader
- CPR coach
- Providing ventilation
- Respiratory therapy responsibilities other than providing ventilation, including initiating end-tidal CO2 monitoring
- Defibrillate the patient
- Chest compressions
- Medication administration
- Documentation
- Other

**Supplemental Figure 1. Distribution of Likert Scale Responses Across Intervention Measures**

IAM: Intervention Appropriateness Measure; AIM: Acceptability of Intervention Measure; FIM: Feasibility of Intervention Measure

Each scale included 4 items completed by 107 respondents, yielding 428 item-level responses per scale.
